# Publisher Correction: Lithospheric foundering and underthrusting imaged beneath Tibet

**DOI:** 10.1038/s41467-018-05925-8

**Published:** 2018-08-27

**Authors:** Min Chen, Fenglin Niu, Jeroen Tromp, Adrian Lenardic, Cin-Ty A. Lee, Wenrong Cao, Julia Ribeiro

**Affiliations:** 1 318 Keith-Wiess Geology Lab, Department of Earth Science, Rice University, MS 126, 6100 Main Street, Houston, TX 77005 USA; 2 State Key Laboratory of Petroleum Resource and Prospecting, and Unconventional Natural Gas Institute, China University of Petroleum, Beijing, 102249 China; 3 Department of Geosciences, Princeton University, Princeton, New Jersey 08544 USA; 4 Program in Applied and Computational Mathematics, Princeton University, Princeton, New Jersey 08544 USA

Correction to: *Nature Communications* ; 10.1038/ncomms15659; published online 06 June 2017

The original version of the Supplementary Information associated with this Article contained an error in Supplementary Figure 4 in which the colours on the maps rendered incorrectly. The correct version of Supplementary Fig. 4 is:

which replaces the previous incorrect version: The HTML has been updated to include a corrected version of the Supplementary Information.

