# Supplementary material for: Lithospheric foundering and underthrusting imaged beneath Tibet
Source: Nat Commun. 2017 Jun 6;8:15659. doi: 10.1038/ncomms15659 (PMC5467168; doi:10.1038/ncomms15659)
Supplement: Supplementary Information — Supplementary Figures and Supplementary References [file ncomms15659-s1.docx]

**
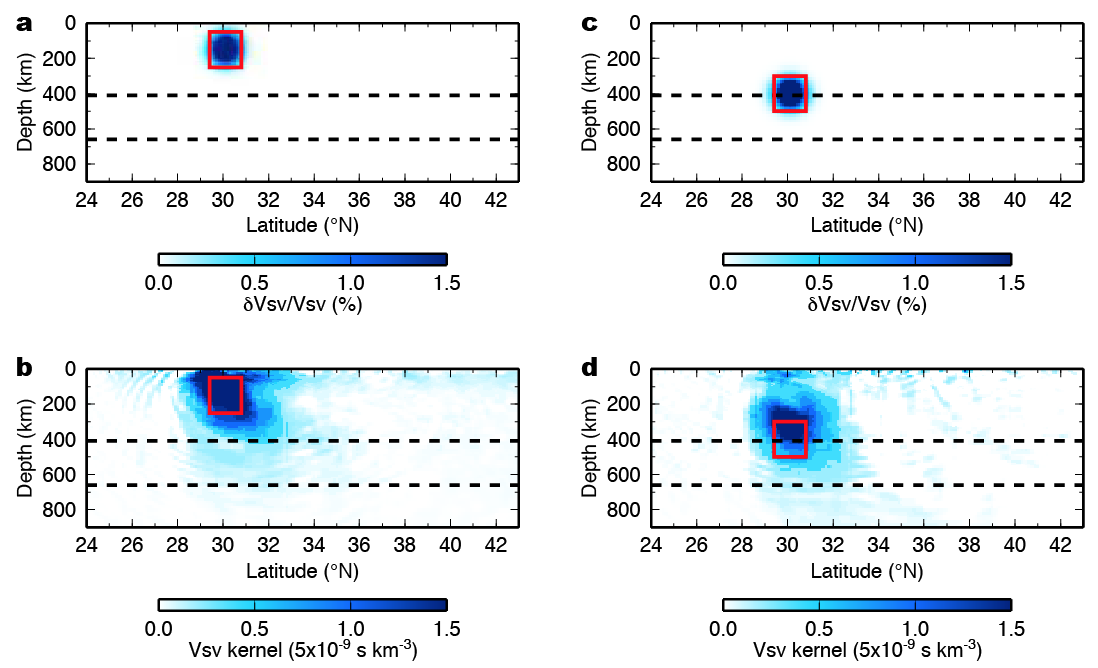
**

**Supplementary Figure 1 | Resolution tests of under Central Tibet.** A 3-D Gaussian spherical perturbation in with a maximum strength of 4% at the center is placed at two different depths, (**a**), 150 km and (**c**), 400 km, in the cross section along profile C (Figs 1 and 4**e**). (**b**) and (**d**) are corresponding Hessian kernel cross sections showing imaging resolution of for its perturbations indicated in **a** and **c** respectively. Red boxes are the visual references for model perturbation and image resolution.

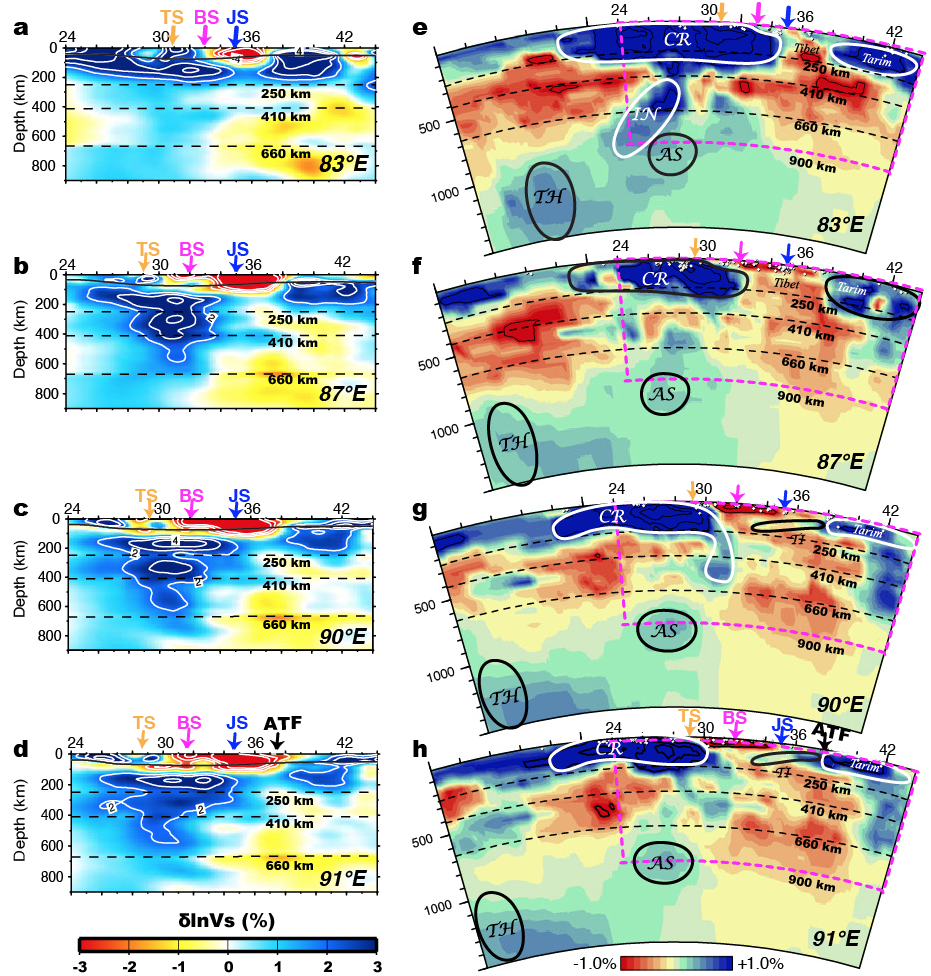


**Supplementary Figure 2 | Comparison between two tomographic models.** (**a**)–(**d**) Cross sections of isotropic shear wave speed anomalies of EARA2014^1^ along longitudes 83°E, 87°E, 90°E, and 91°E respectively. (**e**)–(**h**) Cross sections of P wave speed anomalies from the global P-wave model used in *Replumaz et al.* (2014)^2^ (after their Fig. 3). In **a**–**d**, white lines represent contour levels from −4% to −2% and from 2% to 4% at 1% intervals, black dashed lines mark a depth of 250 km and the 410- and 660-discontinuities, and black solid lines delineate the Moho from CRUST2.0. In **e**–**h**, TH is interpreted to be related to the Tethyan oceanic slab, IN to Indian continental slab, CR to Indian Craton, and AS and TI to Asian continental slabs^2^. For reference, annotations of TS (Tsangpo Suture), BS (Bangong Suture), JS (Jinsha Suture), and ATF (Altyn Tagh Fault) in the P-wave model cross sections (**e**–**h**) are also plotted in the cross sections through model EARA2014 (**a**–**d**). The magenta dashed lined boxes in **e**–**h** show the lateral and depth range of model EARA2014 plotted on the left for side-by-side comparison.

**
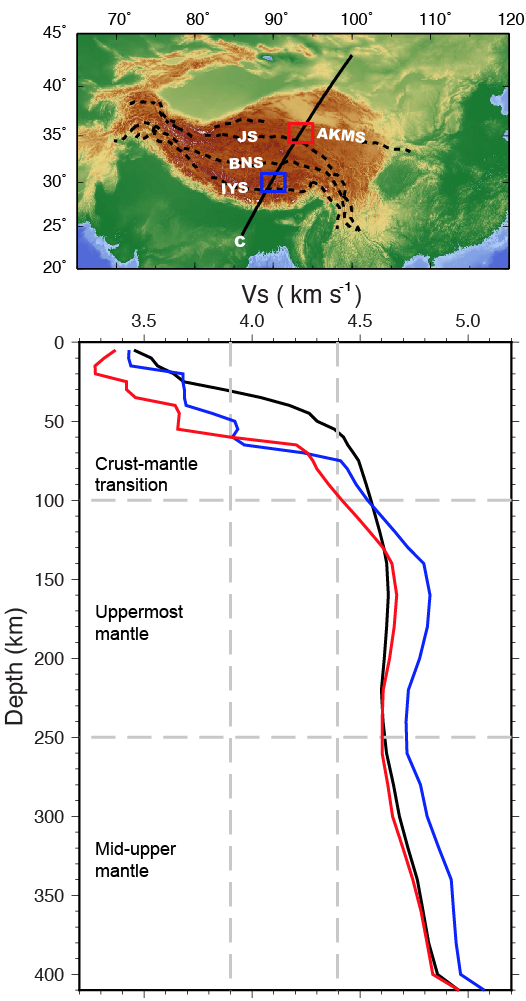
**

**Supplementary Figure 3 | Comparison of average shear wave speed profiles within three regions.** Blue line: profile of the sampling region in Southern Tibet marked by blue box in the map. Red line: profile of the sampling region in Northern Tibet marked by red box in the map. Black line: profile of Tibet and the surrounding region spanning the entire map. Main suture zones and profile C in the map are labeled the same as in Figure 1. Horizontal gray dashed lines indicate 100 km or 250 km depths that define uppermost mantle and mid-upper mantle in this study. Vertical gray dashed lines mark wave speeds of 3.9 km s^−1^ or 4.4 km s^−1^. Regions with wave speeds between 3.9 km s^−1^–4.4 km s^−1^ indicate crust-mantle transition.

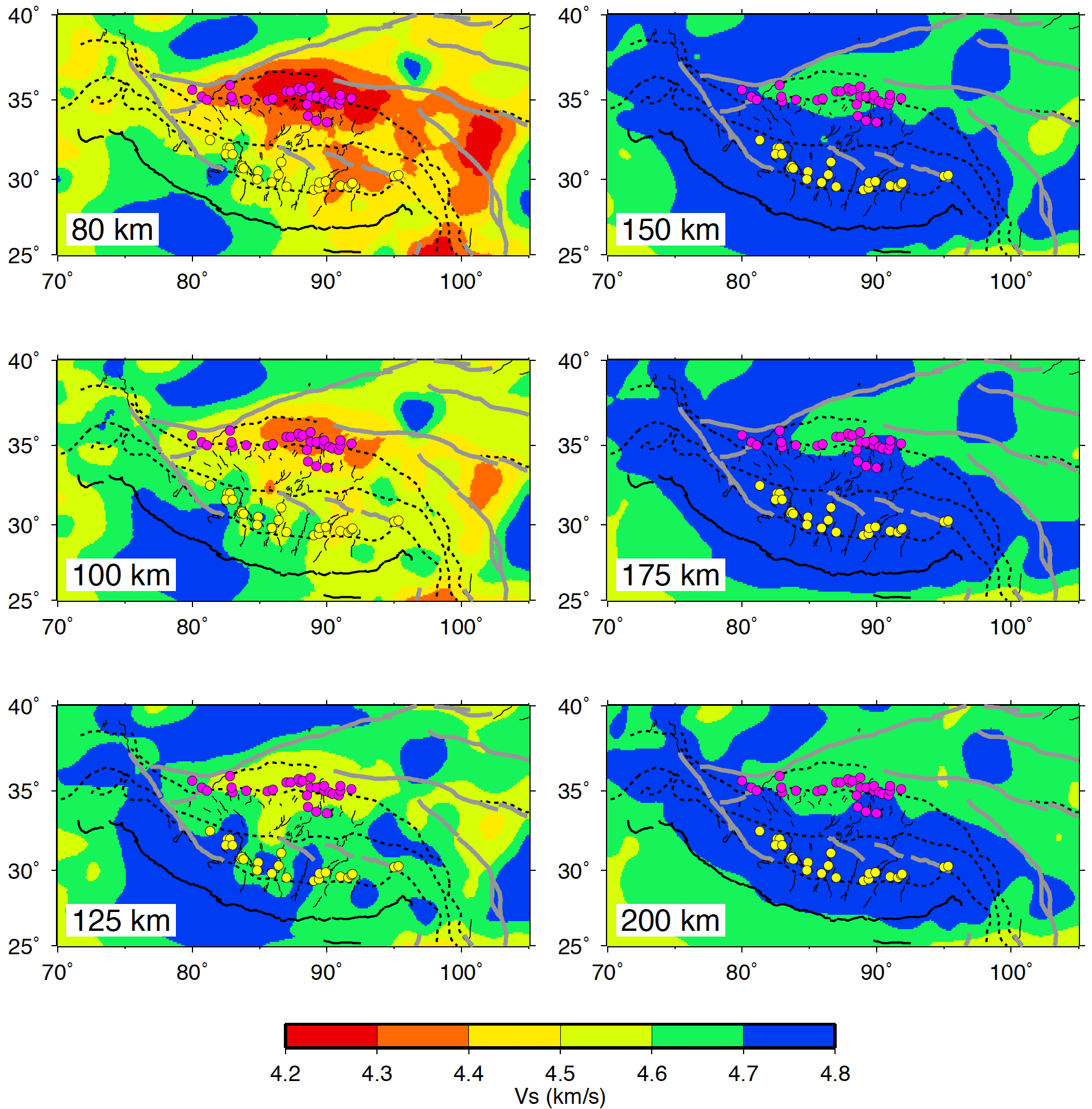


**Supplementary Figure 4 | Maps of absolute shear wave speeds at different depths.**


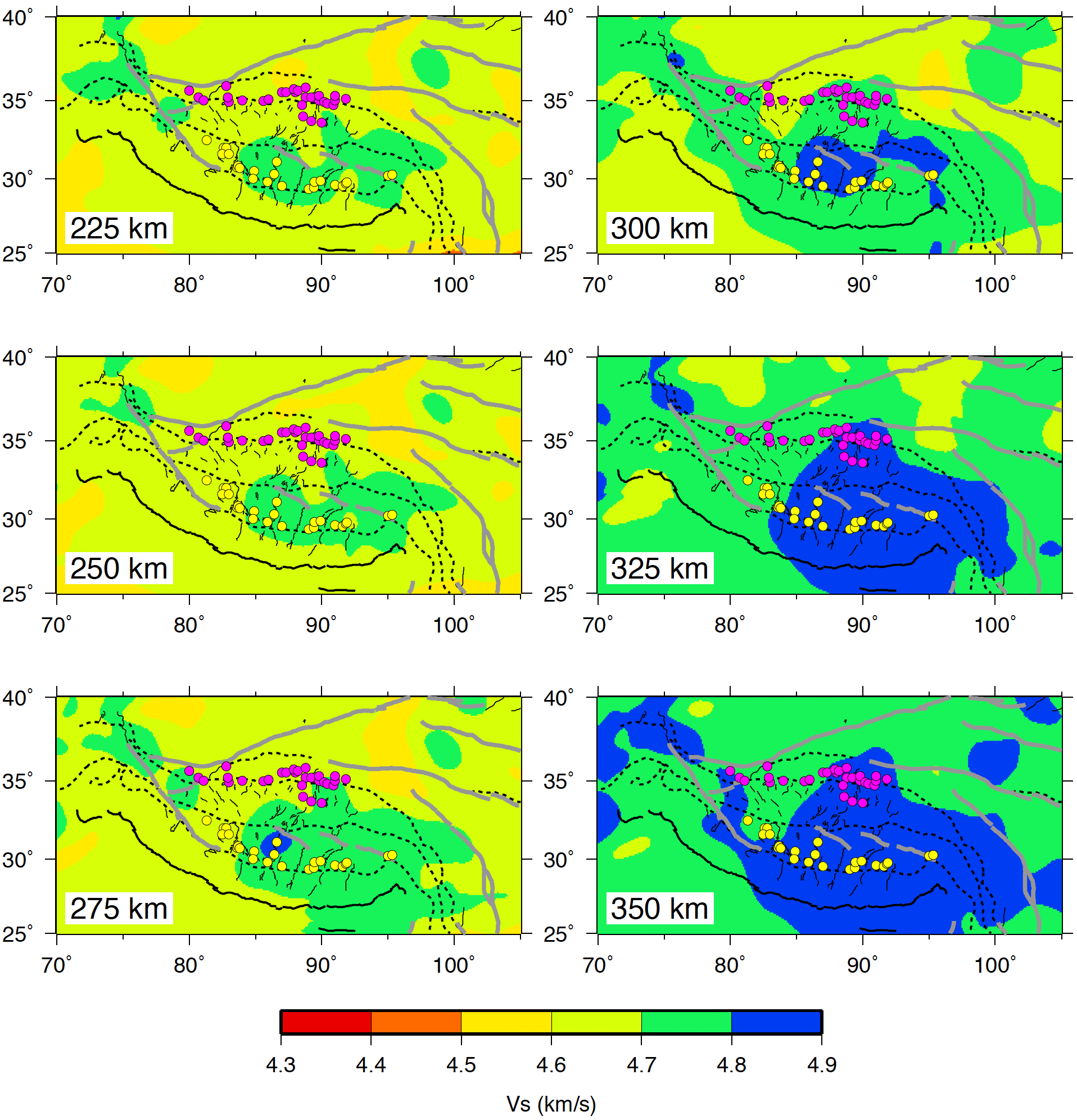


**Supplementary Figure 4 |** (continued)

**
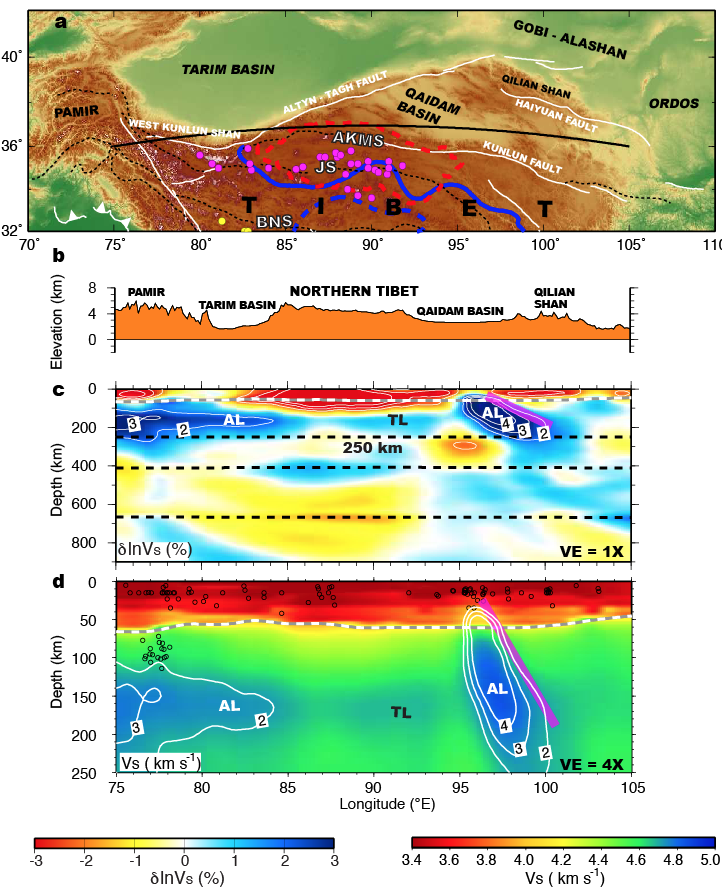
**

**Supplementary Figure 5 | Geological map and cross sections showing surface elevations and seismic structures along latitude 36°N.** (**a**) Geological map of northern Tibet. Map legend is the same as in Figure 1. Thick black line in the map marks the profile location. (**b**) The surface elevations, (**c**) shear wave speed anomalies (), and (**d**) shear wave speeds () along latitude 36°N. In **c**, white lines represent contour levels from −4% to −2% and from 2% to 4% at 1% intervals. In **d**, black circles denote the seismicity, are plotted with 4× of vertical exaggeration (VE = 4×), and white lines represent contour levels from 2% to 4% at 1% intervals extracted from **c**. Thick magenta line in **c** and **d** represents the interpreted Qaidam Basin mantle lithosphere upper interface with an eastward dipping angle of 25°. TL: Tibetan lithosphere. AL: Asian mantle lithosphere under either the Tarim or Qaidam Basins.

**Supplementary References**

1. Chen, M., Niu, F., Liu, Q., Tromp, J. & Zhen, X. Multiparameter adjoint tomography of the crust and upper mantle beneath East Asia: 1. Model construction and comparisons. *J. Geophys. Res.* **120,** 1762–1786 (2015).

2. Replumaz, A., Capitanio, F. A., Guillot, S., Negredo, A. M. & Villaseñor, A. The coupling of Indian subduction and Asian continental tectonics. *Gondwana Res.* **26,** 608–626 (2014).
